# Antimicrobial dispensing process in community pharmacies: a scoping review

**DOI:** 10.1186/s13756-022-01157-0

**Published:** 2022-09-17

**Authors:** Elindayane Vieira de Souza, Lara Joana Santos Caxico Vieira, Sylmara Nayara Pereira dos Santos, Sabrina Cerqueira-Santos, Kérilin Stancine Santos Rocha, Divaldo Pereira de Lyra

**Affiliations:** 1 Graduate Program in Pharmaceutical Sciences, Laboratory of Teaching and Research in Social Pharmacy (LEPFS), Department of Pharmacy, Federal University of Sergipe, Avenue Marechal Rondon, Jd. Rosa Elze, São Cristóvão, Sergipe State 49100-000 Brazil; 2 Laboratory of Teaching and Research in Social Pharmacy (LEPFS), Department of Pharmacy, Federal University of Sergipe, Avenue Marechal Rondon, Jd. Rosa Elze, São Cristóvão, Sergipe State 49100-000 Brazil; 3 Health Sciences Graduate Program, Laboratory of Teaching and Research in Social Pharmacy (LEPFS), Department of Pharmacy, Federal University of Sergipe, Avenue Marechal Rondon, Jd. Rosa Elze, São Cristóvão, Sergipe State 49100-000 Brazil

**Keywords:** Antimicrobials, Dispensing, Community pharmacy, Pharmacists

## Abstract

**Background:**

Antimicrobial resistance remains a major global public health concern, and antimicrobial dispensing in community pharmacies is an important factor in preventing this damage. However, the current literature focuses on the technical and attitudinal aspects related to antimicrobial dispensing, with little emphasis on the interventions provided in this service. Thus, this study aimed to determine the antimicrobial dispensing process in community pharmacies.

**Methods:**

A scoping review was performed in September 2020 using the PubMed, EMBASE, LILACS, Web of Science, and Cochrane databases. The search terms included words related to dispensing, antibacterial agents, and pharmacies in various combinations. Two reviewers screened the titles, abstracts, and full-text articles according to the eligibility criteria, and extracted the data. The findings were presented in a descriptive form.

**Results:**

Of the 7713 studies screened, 35 were included, of which 22 (63%) were published in Asia. Most studies followed a cross-sectional design (n = 27), and the simulated patient was the most often used method to assess the antimicrobial dispensing process (n = 22). Moreover, 31 (89%) studies investigated antimicrobial dispensing without prescription, and only four (11%) studies evaluated antimicrobial dispensing with prescription. In the 35 studies, the most frequently asked questions were about drug allergies (n = 19) and patient symptoms (n = 18), and counseling mainly focused on the side effects (n = 14), precautions (n = 14), how to take the medication (n = 12), and duration of medication use (n = 11). Another common intervention was referral (n = 15). Among clinical cases, counseling on medication use occurred often in cases of urinary tract infection (51%) and otitis media (50%).

**Conclusions:**

Antimicrobial dispensing processes have been primarily investigated in low- and middle-income countries, with a focus on dispensing antimicrobials without prescriptions. During the dispensing process, pharmacists mostly posed minimal questions and counseling, highlighting the deficiencies that persist in this practice. Our results indicate the need for multifaceted strategies, such as implementing educational, regulatory or administrative strategies and changes in cultural background, especially in low- and middle-income countries, that aim to reduce indiscriminate use of antimicrobials. Therefore, qualifying the antimicrobial dispensing process is a fundamental factor for improving the rational use of antimicrobials and reducing microbial resistance.

**Supplementary Information:**

The online version contains supplementary material available at 10.1186/s13756-022-01157-0.

## Background

The development of antimicrobials has been one of the most significant events in modern medicine over the past century [1]. However, their indiscriminate use has become a growing public health concern as antimicrobials have been strongly implicated in the development of microbial resistance [2, 3]. Globally, microbial resistance is responsible for an estimated 700,000 deaths per year, which could increase to as much as 10 million by 2050 if this problem is not addressed [4, 5]. In the United States, microbial resistance has caused an estimated two million infections and 23,000 deaths, with an annual economic impact of US$ 55–70 billion [6].

In this context, health systems worldwide face the challenge of addressing microbial resistance. According to the World Health Organization, approximately 93% of access to antimicrobials comes from community pharmacies [7]. From this perspective, pharmacists can be considered the last barrier during the dispensing process, capable of preventing inappropriate use of antimicrobials and the possible health problems that could result from such use [8]. The dispensing has wide visibility in community pharmacies, is highly accessible, and serves many patients seeking treatment-related counseling and medicine [9].

Drug dispensing is a service that ensures the provision of medicines and other health products through analysis of the technical and legal aspects of a prescription, assessment of individual health needs, and medical intervention through pharmaceutical counseling and documentation [10, 11]. A well-structured dispensing process can become a valuable service because it can limit indiscriminate antimicrobial use, and therefore, microbial resistance [12].

Studies have indicated that interaction between pharmacists and patients enables interventions that optimize the use of antimicrobials in community pharmacies [13]. Despite this, there is little scientific evidence on the panorama of antimicrobial dispensing practices and interventions provided by pharmacists. Previous studies have focused on the frequency and proportion of the sale of antimicrobials without prescriptions in community pharmacies, and the main diseases, and antimicrobials involved in these practices [13–15], as well as pharmacists’ perceptions of antimicrobial sales without prescription [8, 16].

Thus, there remains a gap in the way this service is provided to patients, with little emphasis placed on the dispensing process; in other words, how the dispensing service has been provided to patients and what pharmacist interventions have been carried out during this service. Therefore, exploring pharmacist interventions will optimization of the antimicrobial dispensing service, and development of strategies that mitigate the burden of microbial resistance and its impacts on society.

Hence, studying antimicrobial dispensing processes in community pharmacies worldwide is important. An understanding of the behaviors, counseling, and interventions provided by pharmacists during antimicrobial dispensing, will help to develop strategies that address the gaps in this service and improve the dispensing process in community pharmacies. Therefore, this study aimed to determine the antimicrobial dispensing process in community pharmacies.

## Methods

### Study design

This scoping review adopted the methodology described in the Joanna Briggs Institute Reviewer’s Manual [17] and was based on the Preferred Reporting Items for Systematic Reviews and Meta-Analyses extension for scoping reviews (PRISMA-ScR) criteria [18]. Scoping Review was performed as the objective of the present study was to map, or identify, the available literature related to the process of dispensing antimicrobials [19].

### Identification and development of research questions

As this review mainly focused on the antimicrobial dispensing process, the research question was “what are the antimicrobial dispensing processes in community pharmacies?” Based on this research question, five areas of interest were identified.

- RQ1: What are the most frequently dispensed classes of antimicrobials?
- RQ2: What are the methods used to evaluate antimicrobial dispensing?
- RQ3: What is the main counseling provided to patients by pharmacists and the pharmacy team during antimicrobial dispensing?
- RQ4: What are the others interventions performed by pharmacists and the pharmacy team for patients during antimicrobial dispensing?
- RQ5: How do studies assess the quality of antimicrobial dispensing?

### Search strategy

A literature search was performed in September 2020 using the following databases: PubMed/MEDLINE, LILACS, Cochrane, Web of Science, and Embase. The search strategies were drafted according to a database protocol using search terms related to dispensing, anti-bacterial agents, and pharmacies and their combinations. The search strategy employed both standardized terms from the controlled vocabulary of the “National Library of Medicines” through the “Medical Subject Headings (MESH)” and non-standard terms to extend the search. The “All fields” search option was used. No date limit was used in the database search and relevant studies published up to September 2020 were identified. The full search is available in the Additional file 1.

### Study selection

Two reviewers (E.V.S. and L.J.S.C.V.) independently screened the search results using the Rayyan tool (http://rayyan.qcri.org) [20], and identified potentially relevant studies based on their titles and abstracts. Relevant studies were read in full and selected according to the eligibility criteria. Disagreements between the two reviewers were resolved by a third reviewer (S.C.S.).

### Eligibility criteria

The study selection criteria were established according to the population-concept-context framework, as recommended by the Joanna Briggs Institute for scoping reviews [17]:

- *Population:* community pharmacists and pharmacy team
- *Concept:* antimicrobial dispensing process
- *Context:* community pharmacy

#### Inclusion criteria

Studies that met the following criteria were included: (i) original articles; (ii) studies published in English, Portuguese, and Spanish; (iii) studies exploring antimicrobial dispensing processes; (iv) studies performed in community pharmacies; (v) studies that evaluated interventions during dispensing; and (vi) studies with pharmacists and pharmacy team as participants. No limitations were applied in terms of the publication year or study design.

#### Exclusion criteria

The following studies were excluded: (i) academic documents such as theses and dissertations; (ii) studies without full text available; (iii) meta-analyses, systematic reviews, narrative reviews, letters, editorials, commentaries, posters, and conference proceedings; and (iv) studies in which separating antimicrobial dispensing data from those of other drug classes was not possible.

### Data extraction

A standardized data graph form was created, and data extraction was performed in duplicate. The initial categories included general study characteristics such as authors, year of publication, country, study objective, study design, and sample. Information was collected on antimicrobial dispensing practices, including antimicrobial dispensing with and without prescription, methods for evaluating the dispensing of antimicrobials and assessing the quality of dispensing, classes of antimicrobials dispensed/studied, and limitations. In the present study, evaluation of dispensing quality focused on the instruments or quality indicators used by the studies that reported minimal counseling or other interventions provided by pharmacists for the rational use of medicines.

### Analysis and presentation of the results

The findings were presented in a descriptive form. Figures and tables were used as appropriate to illustrate or summarize the key findings.

## Results

### Search results

**Fig. 1:**
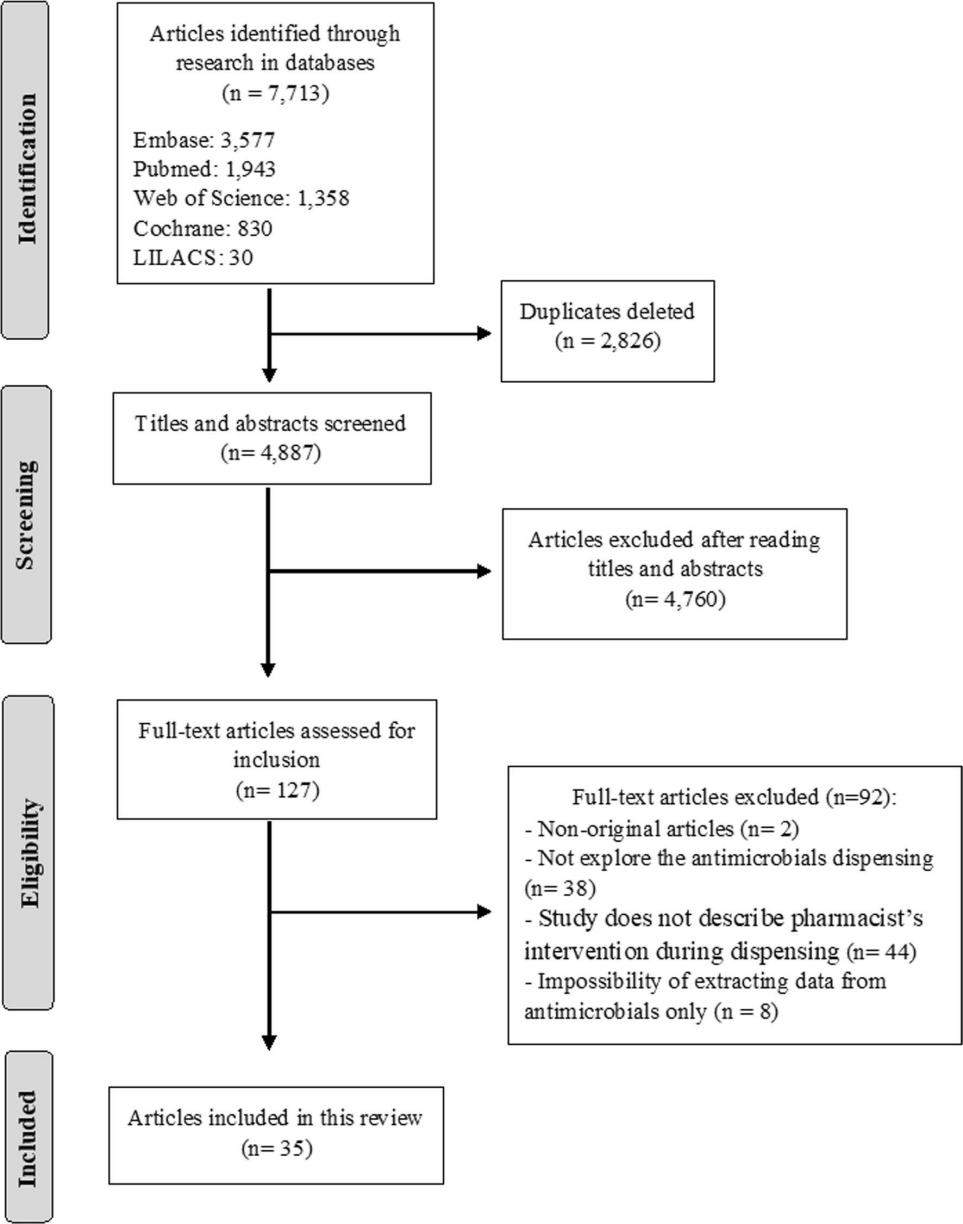
Flow diagram of literature search and screening process

A total of 4887 articles were identified during the initial search. After excluding duplicates (n = 2826) and irrelevant articles based on the titles and abstracts (n = 4760), 127 potentially relevant articles were retrieved for full-text evaluation. Of these, 35 met the inclusion criteria and were included in the scoping review [21–55] (Fig. 1).

### Characteristics of the selected studies

A description of the general characteristics of the 35 included studies is provided in Table 1. These studies were published between 2006 and 2020, and 22 (63%) were conducted in Asia, 7 (20%) in Africa, 4 (11%) in Europe, 1 (3%) in North America, and 1 (3%) in South America. Regarding study design, 31 studies were cross-sectional, 3 were qualitative, and 1 used more than one study design (mixed methods). Moreover, 31 (89%) studies investigated the practice of antimicrobial dispensing/sales without prescription, and only four (11%) studies evaluated the same with prescriptions. We did not observe any difference in the antimicrobial dispensing process with or without prescriptions.

**Table 1:** Characteristics of the selected studies

Study	Country	Aims/objective	Design
Abdelaziz et al. [18]	Egypt	To examine antibiotic dispensing patterns in Egyptian community pharmacies	Cross-sectional
Abubakar and Tangiisuran [19]	Nigeria	To evaluate knowledge, perception, and practices of pharmacists towards dispensing antibiotics without prescription	Cross-sectional
Abuirmeileh et al. [30]	Jordan	To investigate the common practice of dispensing ABs without prescription in community pharmacies	Qualitative
Abujheisha and Ahmed [41]	Saudi Arabia	To estimate the pharmacists who tend to dispense antibiotics without a prescription; and the factors associate	Cross-sectional
Alabid et al. [47]	Malaysia	To explore, assessed and compared dispensing of antibiotics between Pharmacist and General Practitioners	Cross-sectional
Almaaytah et al. [48]	Jordan	To assess of pharmacies that dispense antibiotics without a prescription and identify the seriousness of such practices	Cross-sectional
Al-Tannir et al. [49]	Saudi Arabia	To assess of non-prescribed antibiotic sales by pharmacies and compare it with the findings from our 2011 study	Cross-sectional
Amirthalingam et al. [50]	Malaysia	To assess Pharmacists’ Perceptions and Experiences of Antibacterial Drug Dispensing in Community Pharmacy	Cross-sectional
Bahnassi [51]	Saudi Arabia	To investigate pharmacists’ practices through conducting, including direct questions and hypothetical scenarios	Qualitative
Bahnassi [52]	Syria	To investigate the Syrian pharmacists’ attitudes and practices regarding antibiotic dispensing without prescription	Qualitative
Beaucage et al. [20]	Canada	The impact of a pharmacist telephone follow-up on clinical, costs for patients undergoing antibiotic treatment	Cross-sectional
Bin Abdulhak et al. [21]	Saudi Arabia	To determine the pharmacies who sell antibiotics without prescriptions, examining the potential risks of such practice	Cross-sectional
Chang et al. [22]	China	To quantify sales of antibiotics without a prescription and to assess the quality of services in community pharmacies	Cross-sectional
Chang et al. [23]	China	To quantify non-prescription dispensing of antibiotics at community pharmacies	Cross-sectional and longitudinal
Chowdhury et al. [24]	Bangladesh	To evaluate the impact of an educational intervention to promote guidelines for better acute respiratory illness	Cross-sectional
Contopoulos-Ioannidis et al. [25]	Greece	To evaluate the extent of and factors that determine the inappropriate use of antibiotics without a prescription	Cross-sectional
Erku and Aberra [26]	Ethiopia	To document motivations behind non-prescribed sale of antibiotics among community medicine retail outlets	Cross-sectional
Guinovart et al. [27]	Spain	To evaluate the information provided by the staff of the pharmacy to a simulated patient requesting an antibiotic	Cross-sectional
Hadi et al. [28]	Saudi Arabia	To evaluate knowledge, attitude, and practices of pharmacists towards dispensing antibiotics without prescription	Cross-sectional
Halboup et al. [29]	Yemen	To assess the practice of community pharmacists regarding dispensing antibiotics without a prescription	Cross-sectional
Hallit et al. [31]	Lebanon	To assess practices of pharmacists towards prescribing or dispensing antibiotics without prescription to children	Cross-sectional
Horumpende et al. [32]	Tanzania	To Assess antibiotic dispensing practices by community pharmacy and recommend interventions to improve practice	Cross-sectional
Hoxha et al. [33]	Albanian	To evaluate pharmacists’ tendency to dispense antibiotics without prescription	Cross-sectional
Kalungia et al. [34]	Zambia	Ascertain the extent of non-prescription sales and dispensing of antibiotics in community pharmacies	Cross-sectional
Koji et al. [35]	Ethiopia	To determine the rate of over-the-counter dispensary of antibiotics for common childhood illnesses	Cross-sectional
Llor and Cots [36]	Spain	To quantify the percentage of pharmacies selling antibiotics without an official medical prescription	Cross-sectional
Mansour and Al-Kayalib [37]	Syria	To explore pharmacists’ knowledge, attitudes, and dispensing habits with respect to antibiotics and microbial resistance, in view of the potential link between these and the practice of dispensing of antibiotics without a medical prescription	Cross-sectional
Nyazema et al. [38]	Zimbabwean	To assess the quality of private pharmacy practice with a focus on the extent of antibiotic sales without prescription	Cross-sectional
Puspitasari et al. [39]	Indonesian	To quantify antibiotics sales without a prescription and to explore provision of patient assessment and medicine information related to antibiotics requested with or without a prescription	Cross-sectional
Rauber et al. [40]	Brazil	To evaluate the antibiotics dispensation	Cross-sectional
Shet et al. [42]	India	To determine prevalence of non-prescription sale of antimicrobial drugs by pharmacies	Cross-sectional
Shi et al. [43]	China	To assess non-prescription antibiotic dispensing and assess pharmacy service practice at community pharmacies	Cross-sectional
Yaacoub et al. [44]	Lebanon	To evaluate the antibiotic prescribing rate for acute bacterial rhinosinusitis in community pharmacies and to study the corresponding attitude and behavior of participants	Cross-sectional
Zawahir et al. [45]	Sri Lankan	To evaluate the response of community pharmacy staff to an antibiotic product request without a prescription and to explore possible factors influencing such practice	Cross-sectional
Zawahir et al. [46]	Sri Lankan	To assess responses of community pharmacy staff to pseudo-patients presenting with symptoms of common infections and factors associated with such behaviour	Cross-sectional

### Classes of antimicrobials most often dispensed/studied

Among the classes of antimicrobials most addressed by the studies, were penicillins (n = 54; 33.1%), with amoxicillin and amoxicillin + clavulanate being the most recurring regimens, followed by macrolides (n = 26; 15.9%), cephalosporins (n = 25; 15.3%), quinolones (n = 25; 15.3%), lincosamides (n = 9; 5.5%), aminoglycosides (n = 6; 3.7%), tetracyclines (n = 6; 3.7%), sulfonamides (n = 6; 3.7%), and others (n = 6; 3.7%) (Table 2).

**Table 2:** Antimicrobials dispensing practices of studies

Author, year [References]	Presc	Methods	Scenarios (n)	Sample	Dispenser	Counseling/interventions	Limitations
Abdelaziz et al. [18]	−	Simulated patient and interview	Case 1: Acute Bronchitis (125) Case 2: Common Cold (113)	150 community pharmacies	Pharmacists and attendants	*(Simulated patient) Asked about:* Patient’s identification—case 1: 36.8% (n = 46); case 2: 37.2% (n = 42); Doctor visit or prescription—case 1: 3.2% (n = 1); case 2: 3.4% (n = 4); Patient’s condition—case 1: 0,8% (1); Symptoms—case 1: 35.2% (n = 44); case 2: 36.3% (n = 41); Drug allergy—case 1: 1.6% (n = 2); case 2: 0.9% (n = 1) *(Interview) Asked about:* Doctor visit or prescription—33.9% (n = 20); Patient’s condition—86.4% (n = 51); Symptoms 66.1% (n = 39) *Others interventions:* Prescription of OTC—case 1: 2.4% (7); case 2: 3.6 (4);	N/R
Abubakar and Tangiisuran [19]	−	Questionnaire	N/A	98 community pharmacist	Pharmacists	*Asked about:* Drug allergy—93.9% (n = 92); *Counseling on:* Side effects—79.6% (n = 78); Contact pharmacist/doctor if necessary—94.9% (n = 93); Medication adherence—94.9% (n = 93) *Others interventions:* Referral—69.4% (n = 68); Prescription of OTC—28.6% (n = 28); Health education—92.9% (n = 91)	Social desirability bias Didn’t evaluate pharmacists asked their patients about other medications
Abuirmeileh et al. [30]	−	Questionnaire	N/A	54 community pharmacies	Pharmacists	*Asked about:* Patient’s condition—70% (n = 38); The use of other medicines—92% (n = 50); Drug allergy—94% (n = 51); *Counseling on:* Precautions—90% (n = 49)	N/R
Abujheisha and Ahmed [41]	−	Questionnaire	N/A	155 community pharmacies	Pharmacists	*Asked about:* Drug allergy—91.8% (n = 135); Patient’s condition—87.8% (n = 129); The use of other medicines—90.4% (n = 133); *Counseling on:* Side effects—Always 90.4% (n = 133); Medication adherence—Always 94.5% (n = 139), *Others interventions:* Antimicrobial not dispensed—96.6% (n = 142)	N/R
Alabid et al. [47]	−	Simulated patient	Case 1: Common cold;	100 visits to 50 pharmacies	Pharmacists	*Asked about:* Patient’s identification—Case 1: 12% (n = 12); Patient’s symptoms—Case 1: 87% (n = 87); The use of other medicines—Case 1: 10% (n = 10); Drug allergy—Case 1: 37% (n = 37)	Small sample size Diagnosis and prescription can differ
Almaaytah et al. [48]	−	Simulated patient	Case 1: Sore throat (41); Case 2: Acute sinusitis (39); Case 3: Otitis media (38); Case 4: Diarrhea (42); Case 5: Urinary tract infection (42)	202 community pharmacies	Pharmacists	*Asked about:* Drug Allergy—Case 1: 45% (n = 18); Case 2: 26.7% (n = 4); Case 5: 2.9% (n = 1); The use of other medicines—Case 1: 2.5% (n = 1); Case 3: 15.4% (n = 4); Case 4: 2.9% (n = 1); Case 5: 2.9% (n = 1) *Counseling on:* How to take the medicine—Case 1: 87.5% (n = 35); Case 2: 93.3% (n = 14); Case 3: 100% (n = 26); Case 4: 100% (n = 34); Case 5: 97.1% (n = 34); How long the medicine should be taken—Case 1: 17.5% (n = 7); Case 2: 6.7% (n = 1); Case 3: 11.5% (n = 3); Case 4: 20.6% (n = 7); Case 5: 20% (n = 7); *Others interventions:* Referral—Case 3: 15.4% (n = 4); Case 4: 2.9% (n = 1); Case 5: 2.9% (n = 1)	N/R
Al-Tannir et al. [49]	−	Simulated patient	Case 1: Sore throat (58); Case 2: Acute sinusitis (56); Case 3: Otitis media (54); Case 4: Acute bronchitis (51); Case 5: Diarrhea (57); Case 6: Urinary tract Infection (51)	327 community pharmacies	Pharmacists	*Asked about:* Patient’s symptoms—Case 1: 43.1% (n = 25); Case 2: 66.1% (n = 35); Case 3: 33.3% (n = 18); Case 4: 52.9% (n = 27); Case 5: 54.4% (n = 31); Case 6: 39.2% (n = 20); Drug allergy—Case 3: 1.9% (n = 1); Case 4: 2% (n = 1); Case 5: 1,8% (n = 1); Case 6: 2% (n = 1) *Counseling on:* Precautions—Case 6: 9.8% (n = 5); Side effects—Case 4: 33,3% (n = 17); Case 5: 1,8% (n = 1); Case 6: 5,9% (n = 3) *Others interventions:* Referral—Case 1: 72.4% (n = 42); Case 2: 42.9% (n = 74); Case 3: 88.9% (n = 48); Case 4: 68.6% (n = 35); Case 5: 13.3% (n = 7); Case 6: 80.4% (n = 41)	N/R
Amirthalingam et al. [50]	−	Questionnaire	N/A	101 community pharmacist	Pharmacists	*Asked about:* Patient’s symptoms—86.4% (n = 89); The use of other medicines—71.8% (n = 74); Patient’s condition—64.1% (n = 66); *Counseling on:* Drug indication—95.1% (n = 98); How to take the medicine—94.2% (n = 97); Medication adherence—68.9% (n = 71); Contact pharmacist/doctor if necessary—82.5% (n = 85); Precautions—91.3% (n = 94)	The sample size small; Comparisons were not carried
Bahnassi [51]	−	Semi structured interview	N/A	150 community pharmacist	Pharmacists	*Counseling on:* Antimicrobial not dispensed—63% (n = 94); Side effects—33% (n = 49); *Others interventions:* Modify the antimicrobial—43% (n = 64)	Pharmacists’ self-reporting The interviews were interrupted by customers
Bahnassi [52]	−	Semi-structured interviews	N/A	350 community pharmacies	Pharmacists	*Asked about:* Patient’s condition—36% (n = 126) *Counseling on:* Dosing directions—34% (n = 119) Side effects—47% (n = 165) Modify the antimicrobial—37% (n = 130)	-Pharmacists’ discussion could be biased The low number of participants
Beaucage et al. [20]	+	Telephone follow-up	Case 1: Lower respiratory tract (42); Case 2: Upper respiratory tract (38)	6 community pharmacies	Pharmacists	*Counseling on:* Precautions—case 1: 28% (n = 22); case 2: 3% (n = 2); Discontinue antimicrobial—case 1: 2% (n = 2); case 2: 1% (n = 1); *Others interventions:* Prescription OTC—case 1: 36% (n = 29); case 2: 5% (n = 4); Modify the antimicrobial frequency—case 1: 13% (n = 10); case 2: 1% (1); Contact pharmacist/doctor if necessary—case 1: 9% (n = 7); case 2: 3%(2) Referral—case 1: 9% (n = 7); case 2. 3% (n = 2); Modify the antimicrobial dosage—case 1: 2% (n = 2); case 2: 2% (n = 2); Modify the antimicrobial—case 1: 2% (n = 2); case 2: 1% (n = 1)	N/R
Bin Abdulhak et al. [21]	−	Simulated patient	Case 1: Sore throat (58); Case 2: Sinusitis (55) Case 3: Otitis (53); Case 4: Bronchitis (44); Case 5: Diarrhea (59);Case6:Urinary infection (58)	327 community pharmacies	Pharmacists	*Asked about:* Patient’s symptoms—Case 1: 40% (n = 23); Case 2: 43% (n = 19); Case 3: 22% (n = 13); Case 4: 62% (n = 36); Case 5: 73% (n = 40); Case 6: 19%(n10) Patient’s condition—Case 1: 9% (n = 5); Case 2: 25% (n = 11); Case 3: 14% (n = 8); Case 4: 14% (n = 8); Case 5: 9% (n = 5); Case 6: 2% (n = 1) *Counseling on:* Precautions—Case 3: 23% (n = 13) *Others interventions:* Referral—Case 1: 5% (n = 3); Case 2: 14% (n = 6); Case 3: 10% (n = 6); Case 4: 3% (n = 2); Case 5: 2% (n = 1); Case 6: 47% (n = 25)	N/R
Chang et al. [22]	−	Simulated patient	Case 1: Pediatric diarrhoea (256); Case 2: Adult acute upper respiratory infection (256)	256 community pharmacies	Pharmacists and attendants	*Asked about:* Patient’s condition—case 1: 40.6% (n = 58); case 2: 80.4% (n = 160); Patient’s symptoms—case 1: 4.2% (n = 6); case 2: 32.2% (n = 64); The use of other medicines—case 1: 2.1% (n = 3); case 2: 6.5% (n = 13); Drug allergy—case 1: 59.4% (n = 85); case 2: 41.2% (n = 82); *Counseling on:* How to take the medicine—case 1: 17.5% (n = 25); case 2: 9.6% (n = 19); *Others interventions:* Referral—case 1: 10.6% (n = 12) Antimicrobial not dispensed—case 1: 12.4% (n = 14); case 2: 24.6%(14)	Not differentiate the drugs and services dispensed by a pharmacist
Chang et al. [23]	−	Simulated patient and practice documentation	Case 1: Paediatric diarrhoea (1554) Case 2: Adult Upper Respiratory Trate Infecction (1896)	2411 community pharmacies	Pharmacists and attendants	*Asked about:* Patient’s condition—Case 1: 64.5% (n = 1554); Case 2: 78.6% (n = 1896); The use of other medicines—Case 1: 9.7% (n = 234); Case 2: 6.6% (159); Patient’s symptoms—Case 1: 18.2% (n = 439); Case 2: 13.4% (n = 323); Drug allergy—Case 1: 16.1% (n = 188); Case 2: 29.2% (n = 494); *Others interventions:* Antimicrobial not dispensed—Case 1: 21.5% (251); Case 2: 23.8% (403) Referral—Case 1: 6.5% (n = 156); Case 2: 3.8% (n = 92)	They did not sample in proportion to population; No explore the effect of possible interventions
Chowdhury et al. [24]	−	Simulated patient	N/R	100 community pharmacies	Pharmacists	*Counseling on:* Precautions—5% (n = 8); *Others interventions:* Referral—44% (n = 44); Prescription of OTC—29% (n = 35);	The field staff might not have represented the subtleties of real-life
Contopoulos-Ioannidis et al. [25]	−	Simulated patient	Case 1: Acute uncomplicated rhinosinusitis with low fever (38.5 °C); Case 2: Acute uncomplicated rhinosinusitis with high fever (40ºC)	102 community pharmacies 98 community pharmacist	Pharmacists	*Asked about:* Patient’s symptoms—Case 1: 30% (n = 15); Case 2: 14% (n = 7); Doctor visit or prescription—Case 1: 20% (n = 10); Case 2: 28% (n = 14); Drug allergy—Case 1. 22% (n = 11); Case 2. 12% (n = 6); Patient’s identification—Case 1: 20% (n = 10); Case 2. 22% (n = 11); Patient’s condition—Case 1: 4% (n = 2); Case 2: 4% (n = 2); The use of other medicines—Case 1: 10% (n = 5); Case 2: 14% (n = 7); *Counseling on;* Dosage—Case 1: 88% (n = 37); Case 2: 85% (n = 29); How long the medicine should be taken—1: 64% (n = 27); 2: 74% (25) *Others interventions:* Referral—Case 1. 35% (n = 17); Case 2: 57% (n = 28); Prescription OTC—Case 1: 45% (n = 22); Case 2: 35% (n = 17)	Study design is limited by the fact that the trial was performed in a specific city in Greece
Erku and Aberra [26]	−	Simulated patient and in-depth interview	Case 1: Acute childhood diarrhea (50); Case 2: Uncomplicated Upper Respiratory Infection (50);	20 community pharmacies	Pharmacists and attendants	*Asked about:* Drug allergy—Case 1: 10.7% (n = 3); Case 2: 14.3% (n = 4); *Counseling on:* How to take the medicine—Case 1: 38% (n = 19); Case 2: 34% (n = 17); Side effects—Case 1: 46.4% (n = 13); Case 2: 28.6% (n = 8); Non-pharmacological—Case 1: 12% (n = 6); Case 2: 12% (n = 12) *Others interventions:* Referral—Case 1: 10% (n = 5); Case 2: 8% (n = 4); Antimicrobial not dispensed—Case 1: 14.3% (4); Case 2: 10.7% (n = 3)	The practice behavior in clinical scenarios may not be generalized Recruited smaller mount of pharmacies
Guinovart et al. [27]	−	Simulated patient	Case 1: Urinary Tract Infection or Sore throat or Acute bronchitis	220 community pharmacies	Pharmacists and attendants	*Asked about:* Drug allergy 26% (n = 31); The use of other medicines 1.7% (n = 2); *Counseling on:* How long the medicine should be taken 95.8% (n = 114); *Others interventions:* Referral 36.1% (43) Antimicrobial not dispensed 9.9% (n = 10)	N/R
Hadi et al. [28]	−	Questionnaire	N/A	200 community pharmacist	Pharmacists	*Asked about:* Drug allergy—Always 76.9% (n = 143), sometimes 15.6% (n = 29); Patient’s condition—Always 70.4% (n = 133), sometimes 16.8% (n = 32); *Counseling on:* Side effects—Always 64.6% (n = 122), sometimes 24.9% (n = 47); Medication adherence—Always 88.9% (n = 168), sometimes 5.8% (n = 11); How to take the medicine—Always 81% (n = 153), sometimes 12.2% (n = 23); *Others interventions:* —Antimicrobial not dispensed—Always 61.4% (n = 116), sometimes 20.1% (n = 38);	Self-administered questionnaires such as the one used in this study are prone to social desirability bias
Halboup et al. [29]	−	Simulated patient	Case 1: Sore throat (199); Case 2: Cough (184); Case 3: Otitis (104); Case 4: Urinary tract infection (96); Case 5: Diarrhea (151)	1000 community pharmacies	Pharmacists	*Asked about:* Patient’s symptoms—Case 2: 83% (n = 166); *Counseling on:* How to take the medicine—Case 1: 86.4% (n = 172); Case 2: 39.1% (n = 72); Case 3: 95.1% (n = 98); Case 4: 88.5% (n = 85); Case 5: 76% (n = 117); How long the medicine should be take—Case 1: 72.9% (n = 145); Case 2: 6.5% (n = 12); Case 3: 95.1% (n = 97); Case 4: 61.2% (n = 90); Case 5: 50.6% (n = 78); Precautions—Case 1: 15.6% (n = 31); Case 2: 10.8% (n = 20); Case 3: 2% (n = 3); Case 5: 11.9% (n = 65)	Qualitative data was not obtained to further identify the factors that influence or result in the findings of the study
Hallit et al. [31]	−	Face-to-face interview and Questionnaire	N/A	202 community pharmacist	Pharmacists	*Counseling on:* Drug preparation instructions—81.2% (n = 164) How to take the medicine—53% (n = 107) Dosage—46.5% (n = 94) How long the medicine should be taken—47.5% (n = 96) Storage—64.4% (n = 130) Precautions—81.2% (n = 164) Medication adherence—32.2% (n = 65);	A selection bias due to the exclusion of parents acquiring antibiotics from places other than pharmacies
Horumpende et al. [32]	±	Simulated patient	Case 1: Cough (16) or Fever (13) or Runny nose (22) or Diarrhoea (15) or Pain urination (16)	82 community pharmacies (26 part I; 56 part II)	Pharmacists and attendants	*Counseling on:* Side effects—84.1% (n = 69) *Others interventions:* Prescription of OTC—25.6% (n = 5); Modify the antimicrobial—6% (n = 5); Antimicrobial not dispensed—no prescription 15.8% (n = 13);	The study was not able to collect data on retailers’ qualifications;
Hoxha et al. [33]	−	Questionnaire	Case 1: “I need to get a package of amoxicillin”	450 community pharmacies	Pharmacists	*Asked about:* Patient’s identification 97.6% (n = 253); Drug allergy 58.5% (n = 189); Symptoms 53.2% (n = 172)	N/R
Kalungia et al. [34]	−	Questionnaire	N/A	73 community pharmacies	Pharmacists and attendants	*Asked about:* Drug indication—94.5% (n = 69); *Counseling on:* Dosage—95.9% (n = 49); Side effects—30.1% (n = 22); *Others interventions:* Modify the antimicrobial—97.3% (n = 71)	Simulated patients could have been used but for this was difficult within available resources
Koji et al. [35]	−	Simulated patient	Case 1: Common Cold or Diarrhea or Pneumonia; or Meningitis;	262 community pharmacies	Pharmacists and attendants	*Asked about:* Doctor visit or prescription—62.6% (n = 164); Drug allergy—11.1% (n = 29); Patient’s symptoms—40.8% (n = 107)	N/R
Llor and Cots [36]	−	Simulated patient	Case 1: Urinary tract infection—(69); Case 2: Sore throat (69); Case 3: Acute bronchitis (59);	197 community pharmacies	Pharmacists and attendants	*Asked about:* Patient’s symptoms—case 1: 69.1% (n = 38); case 2: 70.8% (n = 17); case 3: 60% (n = 6); Drug allergy—case 1: 9.1% (n = 5); case 2: 33.3% (n = 8); case 3: 20% (n = 2); Contraindications—case 1: 3.6% (n = 2); *Counseling on:* How to take the medicine—case 1: 94.5% (n = 52); case 2: 70.8% (n = 17); case 3: 50% (n = 5) How long the antibiotic should be taken—case 1: 94.5% (n = 52); case 2: 37.5% (n = 9); case 3: 10% (n = 1); *Other interventions:* Contact pharmacist/doctor if necessary—case 1: 1.8% (n = 1); case 2: 12.5% (n = 3) Antimicrobial not dispensed—case 1: 20.3% (n = 14); case 2: 65.2% (n = 45); case 3: 83.1% (n = 49)	Not distinguish whether the person who attended to the patient was a pharmacist
Mansour and Al-Kayali [37]	−	Questionnaire	N/A	250 community pharmacies	Pharmacists	*Counseling on:* Medication adherence—Always 62.4% (n = 108), sometimes 19.6% (n = 34); Side effects—Always 47.4% (n = 82), sometimes 14.4% (n = 25); Precautions—Always 59% (n = 102), sometimes 18.5% (n = 32); Health education—Always 51.4% (n = 89), sometimes 21.4% (n = 37);	There is the possibility that participants may over-report desirable behaviors
Nyazema et al. [38]	-	Simulated patient and Interviews	Case 1: Vaginal discharge and itching Case 2: Urethral discharge Case 3: A child with acute diarrhoea	44 community pharmacies	Pharmacists	*Asked about:* The use of other medicines—Case 1: 18% (n = 10); Case 2: 3% (n = 2); Patient’s symptoms—Case 1: 91% (n = 52); Case 2: 33% (n = 21) *Counseling on:* Non-pharmacological—Case 3: 37% (n = 25) Side effects—Case 3: 2% (n = 1) Precautions—Case 1: 19% (n = 11) *Others interventions:* Prescription of OTC—Case 1: 58% (n = 33); Case 3: 87% (n = 58);	Repeated visits by simulated clients would have been preferable
Puspitasari et al. [39]	±	Simulated patient	Patient requestes: Case 1: Ciprofloxacin 500 mg Case 2: Tetracycline 250 mg Case 3: Amoxicillin dry syrups 125 mg per 5 ml	105 community pharmacies	Pharmacists and attendants	*Asked about:* Patient’s identification—Case 1: 2% (n = 2); Case 2: 2% (n = 2); Case 3: 31% (n = 23); Patient’s symptoms—Case 1: 2% (n = 2); Case 2: 8% (n = 7); Case 3: 5%(4) *Counseling on:* How to take the medicine—Case 1: 35% (n = 31); Case 2: 68% (n = 60); Case 3: 70% (n = 52); How long the medicine should be taken—Case 1: 21% (n = 18); Case 2: 6% (n = 5); Case 3: 43% (32); Side effects—Case 1: 1% (n = 1); Precautions—Case 1: 1% (n = 1); Medication adherence—Case 1: 2% (n = 2); Case 3: 1% (n = 1); Storage—Case 3: 14% (n = 10)	Data on pharmacy staff’s qualifications were based on self-report of respondents
Rauber et al. [40]	−	Questionnaire	N/A	46 community pharmacist	Pharmacists	*Asked about:* Drug allergy—15.1% Patient’s condition—15.1% Patient’s identification—1% *Counseling about:* How long the medicine should be taken—1.8% Drug interactions—12.6%; Side effects—7.2%; Posology—46.7% *Others interventions:* Antimicrobial not dispensed—1.8%	Relied on data reported by the respondents
Shet et al. [42]	−	Simulated patient	Case 1: Upper respiratory tract infection in adult (115); Case 2: Acute gastro-enteritis in child (146);	261 community pharmacies	Pharmacists	*Counseling on:* Dosage—Case 1: 96.3% (n = 79); Case 2: 23.9% (n = 22); How long the medicine should be taken—Case 1: 91.5% (n = 75); Case 2: 15.2 (n = 14); Non-pharmacological—Case 1: 18.3% (n = 21); Case 2: 12.3% (n = 18); *Others interventions:* Referral—Case 1: 21.7% (n = 25); Case 2: 33.6% (n = 49); Antimicrobial not dispensed—Case 1: 9.1% (n = 3); Case 2: 24.1% (13);	Did not distinguish whether the dispensing workforce in pharmacies
Shi et al. [43]	−	Simulated patient	Case 1: Adult acute cough associated with a common cold (n = 100); Case 2: A pediatric acute cough associated with a common cold (n = 81)	147 community pharmacies	Pharmacists and attendants	*Asked about:* Patient’s symptoms—Case 1: 82.2% (n = 60); Case 2: 82.4% (n = 61); The use of other medicines—Case 1: 82.2% (n = 121); Case 2: 82.4% (61); Doctor visit or prescription—Case 1: 19.2% (n = 14); Case 2: 10.8% (8); Drug allergy—Case 1: 42.5% (n = 31); Case 2: 35.1% (n = 26); *Counseling on:* Non-pharmacological—Case 1: 1.4% (n = 1); 6.8% (n = 5); Side effects—Case 1: 2.7% (n = 2); Case 2: 4% (n = 3); *Others interventions:* Prescription of OTC—Case 1: 21.9% (n = 16); Case 2: 40.5% (30); Referral—Case 1: 5.5% (n = 4)	Did not distinguish whether the respondent was a licensed pharmacist or pharmacy assistant
Yaacoub et al. [44]	±	Simulated patient	Case 1: Bacterial rhinosinusitis	250 community pharmacies	Pharmacists and attendants	*Asked about:* Patient’s identification—19.6% (n = 49); Drug allergy—3.2% (n = 8); Patient’s symptoms—43.2% (n = 108) *Counseling on:* Precautions—2% (n = 5); Drug indication—10.4% (n = 26); *Others interventions:* Referral—10.4% (n = 26)	The sociodemographic characteristics of the participants were not available
Zawahir et al. [45]	−	Simulated patient	Patient requests: Case 1: Erythromycin; Case 2: Amoxicillin Case 3: Metronidazole 500 mg; Case 4: Ciprofloxacin	242 community pharmacies	Pharmacists and attendants	*Asked about:* Patient’s identification—Case 1: 18.3% (n = 11); Case 2: 27.4 (n = 17); Case 3: 17.5% (n = 10); Case 4: 33.3% (21); Patient’s symptoms—Case 1: 1.7% (n = 1); Case 2: 1.6% (n = 1); Case 3: 1.8% (n = 1); The use of other medicines—Case 2: 6.5% (n = 4); Case 3: 1.8% (n = 1); Patient’s condition—Case 1: 6.7% (n = 4); Case 2: 14.5% (n = 9); Case 3: 15.8% (9); Case 4: 11.1% (7); *Others interventions:* Referral—Case 1: 6.7% (n = 4); Case 2: 11.3% (n = 7); Case 3: 1.8% (n = 1); Case 4: 6.3% (4);	Interpersonal variations between SCs impacted how they behaved in the pharmacies, and hence how pharmacy staff behaved
Zawahir et al. [46]	−	Simulated patient	Case 1: Sore throat (60); Case 2: Common cold (60); Case 3: Diarrhea (60); Case 4: UTI (62)	242 community pharmacies	Pharmacists and attendants	*Asked about:* Patient’s symptoms—Case 1: 12% (n = 7); Case 2: 18% (n = 11); Case 3: 10% (n = 6); Case 4: 2% (n = 2); The use of other medicines—Case 1: 1.7% (n = 4); Case 2: 3% (n = 2); Case 3: 2% (n = 1); Case 4: 2% (n = 1); Drug allergy—Case 1: 19% (n = 5); Case 3: 7% (n = 2); Case 4: 9% (n = 3); *Counseling on:* How to take the medicine—Case 1: 62% (n = 16); Case 2: 33% (n = 3); Case 3: 53% (n = 16); Case 4: 71% (n = 24); How long the medicine should be taken—Case 1: 15% (n = 4); Case 3: 23% (n = 7); Case 4: 32% (n = 11); *Others interventions:* Referral—Case 1: 10% (n = 6); Case 2: 23% (n = 14); Case 3: 15% (n = 9); Case 4: 24% (n = 15)	A self- selection of the study participants may have impacted the study findings

NR, Not related; N/A, Not applicable; Presc., Prescription; ( −), Without prescription; ( +), With prescription; ( ±) With and without prescription

### Methods used to evaluate antimicrobial dispensing process

Of the 35 studies included in this review, 22 (63%) used the simulated patient method to evaluate antimicrobial dispensing and 13 (37%) used face-to-face interviews, questionnaires, and pharmacist documentation methods (Table 2). In studies that used the simulated patient method, the main scenarios were related to the following health conditions: sore throat, diarrhea, respiratory tract infection, urinary tract infection, and otitis media. The study samples were heterogeneous and varied from 6 to 2,411 community pharmacies and 98 to 202 pharmacists.

### Antimicrobial dispensing process

Regarding dispensing antimicrobials, community pharmacists and pharmacy team asked questions more frequently than provide counseling or other interventions. Of the 35 studies, the most frequently asked questions were about drug allergies (n = 19, 54.3%), patient symptoms (n = 18, 51.4%), and the use of other medicines (n = 13, 37.1%). The main counseling provided by community pharmacists was about the side effects (n = 14, 40%), and precautions (n = 14, 40%), how to take the medicine (n = 12, 34.3%), and duration of medication (n = 11, 31.4%). In addition, seven other interventions were performed, of which referral was the most common (n = 15, 42.8%), followed by refusal to dispense antimicrobials without prescription (n = 12, 34.3%) (Table 2).

The process of dispensing antimicrobials in clinical cases is presented in Table 3. It was observed that for most cases, the pharmacists asked about patient symptoms, of which cough (78%) and common cold (51%) were the most common. Among clinical cases, counseling on how to take the medicine was most frequent in cases of urinary tract infection (51%) and otitis media (50%), followed by how long the medication should be taken in cases of urinary tract infection (42%) and otitis media (40%). Regarding other interventions, the most frequent was referrals, with the highest frequencies for acute sinusitis (26%) and otitis media (23%). Of note, no study reported the evaluation of antimicrobial dispensing quality.

**Table 3:** Practices of antimicrobials dispensing for clinical cases

Interventions	Diarrhea (n = 2375)	Upper respiratory tract infection (n = 2355)	Sore throat (n = 485)	Acute sinusites (n = 400)	Urinary tract infection (n = 378)	Cough (n = 365)	Acute bronchitis (n = 273)	Common cold (n = 273)	Otitis media (n = 249)	Lower respiratory tract infection (n = 42)
*Asked about*										
Patient’s identification	–	–	–	49 (12%)	–	–	82 (29%)	54 (20%)	–	–
Patient’s condition	1065 (45%)	1650 (70%)	5 (1%)	11 (3%)	1 (0.2%)	–	9 (3%)	–	8 (3.2%)	–
Patient’s symptoms	522 (22%)	387 (16%)	72 (14.8%)	162 (40%)	70 (18.5%)	287 (78%)	77 (27%)	139 (51%)	31 (12%)	–
Drug allergy	279 (12%)	580 (24.6%)	31 (6.4%)	26 (6.5%)	10 (2.6%)	57 (15.6%)	5 (1.8%)	38 (14%)	1 (0.4%)	–
The use of other medicines	239 (10%)	172 (7.3%)	5 (1%)	–	2 (0.5%)	182 (50%)	–	12 (4.4%)	4 (1.6%)	–
Contraindications	–	–	–	–	2 (0.5%)	–	–	–	–	–
Doctor visitor prescription	–	–	–	–	–	16 (4.4%)	–	4 (1.4%)	–	–
*Counseling on*										
Drug preparation instructions	–	–	–	–	–	–	–	–	–	–
Drug indication	–	–	–	26 (6.5%)	–	–	–	–	–	–
Drug interactions	–	–	–	–	–	–	–		–	–
How to take the medicine	192 (8%)	36 (1.5%)	240 (49%)	14 (3.5%)	195 (51%)	72 (19.7%)	5 (1.8%)	3 (1%)	124 (50%)	–
Dosage	22 (0.9%)	79 (3%)	–	–		–	–	–	–	–
How long the medicine should be taken	106 (4.5%)	75 (3.2%)	165 (34%)	1 (0.25%)	160 (42%)	12 (3.3%)	1 (0.3%)	–	100 (40%)	–
Medication adherence	–	–	–	–	–	–	–	–	–	–
Discontinue antimicrobial	–	1 (0.04%)	–	–	–	–	–	–	–	2 (4.8%)
Storage	–	–	–	–	–	–	–	–	–	–
Health education	–	–	–	–	–	–	–	–	–	–
Non-pharmacological measures	24 (1%)	33 (1.4%)	–	–	–	6 (1.6%)	–	–	–	–
Precautions	65 (2.7%)	2 (0.08%)	31 (6.4%)	5 (1.2%)	5 (1.3%)	20 (5.5%)	–	–	16 (6.4%)	22 (52%)
Side effects	14 (0.6%)	8 (0.3%)	–	–	3 (0.8%)	5 (1.3%)	17 (6%)	–	–	–
*Other’s interventions*										
Referral	240 (10%)	94 (4%)	45 (9.3%)	106 (26%)	81 (21%)	4 (1.1%)	37 (13%)	14 (5%)	58 (23%)	7 (16%)
Contact pharmacist/doctor if necessary	–	2 (0.08%)	3 (0.6%)	–	1 (0.2%)		–	–	–	7 (16%)
Modify the antimicrobial	–	1 (0.04%)	–	–	–		–	–	–	2 (4.8%)
Modify the antimicrobial dosage	–	2 (0.08%)	–	–	–		–	–	–	2 (4.8%)
Modify the antimicrobial frequency	–	1 (0.04%)	–	–	–		–	–	–	10 (23.8%)
Prescription of OTC	–	4 (0.17%)	–	–	–	46 (12.6%)	7 (2.5%)	4 (1.4%)	–	29 (69%)
Antimicrobial not dispensed	269 (11%)	423 (18%)	9.3%	–	14 (3.7%)		49 (17%)	–	–	–

The total n of each clinical case is equivalent to the sum of the n of the studies included for each scenario

## Discussion

Despite extensive literature on antimicrobial dispensing, most studies have focused on dispensing antimicrobials without prescriptions. This is especially true in the low- and middle-income countries in Asia and Africa. Corroborating these data, a systematic review by Batista et al. [16] identified that the highest percentage of dispensing antimicrobials without prescription occurred in Asia. Among the main factors that contribute to this practice are the unavailability and inaccessibility of health facilities, economic benefits to the pharmacy team, limited knowledge of antimicrobials among the population and pharmacy team, lack of information about the negative impact of this practice, and ineffectively imposed regulations [16, 56–58]. Therefore, multifaceted strategies should be developed to address these problems, including population awareness and professional qualifications, to reduce inappropriate antimicrobial use.

Most of the studies included in this review used the simulated patient method to assess the antimicrobial dispensing process. The simulated patient method consists of the visit of a trained individual to the pharmacy, indistinguishable from a genuine patient, who enacts predetermined scenarios, to assess information gathering and counseling provided by pharmacists in the supply of medicines. Simulated patient methods provide an unobtrusive means of observing actual practice behavior and are considered the ‘gold standard’ for the measurement of the quality of behavior [59–61]. Furthermore, this method has considerable advantages over other methods for assessing service quality as it minimizes the Hawthorne effect [59]. Thus, studies should use the simulated patient method to assess the care provided by pharmacists and promote a change in practice.

In the present study, pharmacists and pharmacy team asking more questions than provided counseling on medicine use. Other studies have also shown low percentages of counseling provided by pharmacists and pharmacy team [16, 51]. The literature agrees that pharmaceutical counseling is an essential component of dispensing [11, 62, 63]. The counseling provided by pharmacists during dispensing not only reduces inappropriate use of antimicrobials but also improves treatment adherence and increases patient awareness of the importance of the treatment regimen [64]. Therefore, although certain factors can influence the quantity and quality of pharmaceutical counseling, this intervention must always exist in the drug dispensing.

Among the included studies, medical referral was another intervention performed to encourage patients to attend primary healthcare units. Likewise, a study by Chang et al. [51] also noted that in some cases, referrals were recommended instead of dispensing antimicrobials without prescription. Other studies have reported low rates of intervention and interest in patient health needs [42, 65]. Chowdhury et al. [52] conducted educational interventions aimed at controlling the indiscriminate sale of antimicrobials; the interventions led to fewer referrals and only reduced the dispensing of antimicrobials for children. This evidence raises concerns regarding the attitude adopted by pharmacists and pharmacy teams, as medical referrals can potentially reduce the development of microbial resistance owing to the indiscriminate use of antimicrobials for health conditions that do not require such treatment. Thus, there is an urgent need to encourage a proactive attitude among pharmacists and to combine educational interventions by the pharmacy team and raising awareness about proper antimicrobial use among the general population.

This scoping review revealed that no study has assessed the quality of the antimicrobial dispensing process thus far, highlighting an unmet need. Previous studies have used methods to assess dispensing processes, such as the use of instruments available in the literature [65] and performance assessment sheets constructed by the authors based on guidelines and observation protocols by the authors [66]. Although the identification and measurement of quality services is challenging, quality assessment is imperative to identify problems, deficiencies in the provision of care, and points of improvement, and to delineate strategies to overcome these deficiencies and monitor the effectiveness of corrective measures [67]. Therefore, future studies should invest in the development of quality indicators for antimicrobial dispensing and the development and validation of instruments to assess the quality of dispensing. We also recommend that future studies be conducted to provide evidence for the quality of the antimicrobial dispensing process.

In low- and middle-income countries, we noted that properly trained pharmacists and pharmacy team could be a part of the solution to overcome this global challenge of microbial resistance, and we emphasize that training can improve the role of pharmacy professionals [68]. These issues reinforce the need for pharmacists to continue their education while working in community pharmacies. Our findings highlight the urgency for policymakers to develop multifaceted approaches to qualify for the practice of dispensing antimicrobials. Therefore, there is a need for a patient-focused approach that minimizes the sale of these medicines without prescription.

To address the problem of the indiscriminate use of antimicrobials, multifaceted strategies, including implementing educational and/or regulatory/administrative measurements, promoting changes in cultural practices, and adopting advertising resources with the potential for reinforcement and perpetuation of information that aims educate the population about the risks regarding the indiscriminate use of this class of medicines. Moreover, dispensing practice should be regulated, as it has the potential to mitigate the irrational use of antimicrobials and reduce microbial resistance, in view of the strategic position occupied by community pharmacists and pharmacy teams.

### Strengths and limitations

To the best of our knowledge, this scoping review is the first to map and analyze, in the current literature, the range of studies that investigated the dispensing of antimicrobials with and without prescriptions, with a focus on pharmacist interventions. Hence, this study did not use backward or forward snowballing to identify additional relevant studies. There was heterogeneity in the study design, sample, and methods; thus, caution should be exercised when generalizing the results to other health conditions and specific patient populations.

## Conclusion

Antimicrobial dispensing processes have mainly been investigated in low- and middle-income countries, with a focus on dispensing antimicrobials without prescriptions. Minimal questions were asked, and patient counseling was below the levels expected of pharmacists and pharmacy team during the dispensing process, identifying a deficiency in the practice. Therefore, improving antimicrobial dispensing processes to promote the rational use of these medicines is necessary. Furthermore, stakeholders should develop multi-faceted strategies to mitigate microbial resistance.

## Supplementary Information

**Additional file 1.** Database search strategy.

## Data Availability

The datasets used and/or analyzed during the current study are available from the corresponding author upon reasonable request.

## Abbreviations

ABs: Antibiotics
JBI: Joanna Briggs Institute
MeSH: Medical subject headings
N/A: Not applicable
N/R: Not related
OTC: Over-the-counter
PCC: Population-concept-context framework
Presc.: Prescription
PRISMA: Preferred reporting items for systematic reviews and meta-analyses
UTI: Urinary tract infection
