# Supplementary material for: Antimicrobial dispensing process in community pharmacies: a scoping review
Source: Antimicrob Resist Infect Control. 2022 Sep 17;11:116. doi: 10.1186/s13756-022-01157-0 (PMC9482305; doi:10.1186/s13756-022-01157-0)
Supplement: Supplementary file 1 — Additional file 1. Database search strategy. [file 13756_2022_1157_MOESM1_ESM.docx]

**Additional file A - Database search strategy**

| *Pubmed* | #1 | Search: ("Pharmacies"[Mesh]) AND (((((((((("Pharmacists"[Mesh]) OR ("Counseling"[Mesh])) OR ("Anti-Bacterial Agents"[Mesh])) OR ("Dispensing")) OR ("Drug dispensing")) OR ("Drug dispensing practices")) OR ("Drug dispensing practice")) OR ("Antibiotic dispensing practices")) OR ("Antibiotic dispensing practice")) OR ("Antibiotic dispensing")) |
| --- | --- | --- |
|  | #2 | Search: (((((((((("Counseling"[Mesh]) OR ("Anti-Bacterial Agents"[Mesh])) OR ("Dispensing")) OR ("Drug dispensing")) OR ("Drug dispensing practices")) OR ("Drug dispensing practice")) OR ("Antibiotic dispensing practices")) OR ("Antibiotic dispensing practice")) OR ("Antibiotic dispensing")) AND ("Pharmacists"[Mesh])) AND ("Pharmacies"[Mesh]) |
|  | #3 | Search: ((((((((("Counseling"[Mesh]) OR ("Anti-Bacterial Agents"[Mesh])) OR ("Dispensing")) OR ("Drug dispensing")) OR ("Drug dispensing practices")) OR ("Drug dispensing practice")) OR ("Antibiotic dispensing practices")) OR ("Antibiotic dispensing practice")) OR ("Antibiotic dispensing")) AND ("Pharmacies"[Mesh]) |
| *LILACS* | #1 | "Pharmacists" OR "Clinical Pharmacists" OR "Clinical Pharmacist" OR "Pharmacist, Clinical" OR "Pharmacists, Clinical" OR "Community Pharmacists" OR "Community Pharmacist" OR "Pharmacist, Community" OR "Pharmacists, Community" OR "Retail Pharmacists" OR "Pharmacist, Retail" OR "Pharmacists, Retail" OR "Retail Pharmacist" OR "Pharmacist" |
|  | #2 | "Counseling" OR "Dispensing" OR "Drug dispensing" OR "Drug dispensing practices" OR "Antibiotic dispensing practices" OR "Antibiotic dispensing" OR "Antibiotic-dispensing practice" OR "Anti-Bacterial Agents" OR "Anti-Bacterial Agents" OR "Agents, Anti-Bacterial" OR "Anti Bacterial Agents" OR "Antibacterial Agents" OR "Agents, Antibacterial" OR "Anti-Bacterial Compounds" OR "Anti Bacterial Compounds" OR "Compounds, Anti-Bacterial" OR "Bacteriocidal Agents" OR "Agents, Bacteriocidal" OR "Bacteriocides" OR "Anti-Mycobacterial Agents" OR "Agents, Anti-Mycobacterial" OR "Anti Mycobacterial Agents" OR "Antimycobacterial Agents" OR "Agents, Antimycobacterial" OR "Antibiotics" OR "Antibiotic" |
|  | #3 | "Pharmacies" OR "Pharmacy Distribution" OR "Distribution, Pharmacy" OR "Distributions, Pharmacy" OR "Pharmacy Distributions" OR "Community Pharmacies" OR "Community Pharmacy" OR "Pharmacies, Community" OR "Pharmacy, Community" |
